# Childhood Emotional Neglect Is Associated With Low Social Support in Chinese Patients With Major Depressive Disorder

**DOI:** 10.3389/fpsyt.2021.781738

**Published:** 2021-12-02

**Authors:** Xuemei Qin, Mi Wang, Xiaowen Lu, Jinrong Sun, Qiangli Dong, Liang Zhang, Jin Liu, Yumeng Ju, Ping Wan, Hua Guo, Futao Zhao, Yan Zhang, Bangshan Liu, Lingjiang Li

**Affiliations:** ^1^National Clinical Research Center for Mental Disorders, and Department of Psychiatry, The Second Xiangya Hospital of Central South University, Changsha, China; ^2^Mental Health Institute of Central South University, China National Technology Institute on Mental Disorders, Hunan Technology Institute of Psychiatry, Hunan Key Laboratory of Psychiatry and Mental Health, Hunan Medical Center for Mental Health, Changsha, China; ^3^Affiliated WuTaiShan Hospital of Medical College of Yangzhou University, Yangzhou Mental Health Center, Yangzhou, China; ^4^Zhumadian Psychiatric Hospital, Zhumadian, China

**Keywords:** emotional neglect, social support, major depressive disorder, childhood maltreatment, Chinese

## Abstract

**Background:** Previous studies have shown that childhood maltreatment (CM) is closely associated with social support in the general population. However, little is known about the associations of different types of CM with social support in Chinese patients with major depressive disorder (MDD), which was the goal of the current study.

**Methods:** One hundred and sixty-six patients with moderate-to-severe MDD were enrolled. Participants were assessed by the Childhood Trauma Questionnaire-28 item Short Form, Social Support Rating Scale (SSRS), the 24-item Hamilton rating scale for depression, and the 14-item Hamilton Anxiety Rating Scale. Correlation analysis and Hierarchical multiple linear regression analysis were adopted to investigate associations of types of CM with social support.

**Results:** (1) Physical neglect (PN) and emotional neglect (EN) were the most commonly reported types of CM in patients with MDD. (2) EN was the only type of CM significant in the regression models of the SSRS total score, the score of subjective support, and the score of utilization of support.

**Limitations:** The data of CM was collected retrospectively and recall bias may be introduced. Assessment of CM and social support were self-reported and could be influenced by the depression status.

**Conclusion:** In Chinese patients with MDD, PN and EN are the most prevalent types of CM. EN is the only type of CM associated with low social support in regression models, calling for special attention in the assessment and intervention of EN.

## Introduction

Social support is the assistance or comfort from others that makes one person believe himself is a part of a social network, being valued, esteemed, loved, and cared for (1). Social support is associated with the development of depression (2). A high level of support helps individuals to cope with life stress and acts as a protective factor for depression (3). Childhood maltreatment (CM) is defined as a variety of adverse experiences of a child, including emotional abuse (EA), physical abuse (PA), sexual abuse (SA), emotional neglect (EN), and physical neglect (PN) (4, 5). CM also brings detrimental consequences for the mental health of survivors (5, 6), containing earlier onset (7), poorer treatment response (8), and higher risk of relapse (9) of major depressive disorder (MDD).

Considering the both vital role of social support and CM on depression, we were curious about the association of CM with social support. Current literature on this topic is mainly focused on the general population. Generally, CM is found to be correlated with lower levels of social support (10, 11). According to prospective studies, maltreated children are more likely to report lower social support in adulthood (12) and unstable support across the life span (13) compared to those without a history of CM. Specific to types of CM, PA and EA are reported to be correlated with lower support in early life (14) and midlife (15) in the USA. Survivors with childhood neglect have lower social support from their families in Australia (16). In China, Wang et al. found five types of CM are all correlated with social support in adolescents (17) while only EA and PN are related to total social support and objective support, respectively, in a community sample (18).

Up to now, there is no study to investigate this topic in Chinese patients with MDD. As a common, severe, and debilitating neuropsychiatric disorder, MDD will rank first place in the global disease burden by 2030 (19). Investigating associations of CM with social support in MDD may be instructive to the improvement of social support and facilitate the prevention and recovery of depression. Thus, the main objective of the current study is to examine the associations of different types of CM with social support in Chinese patients with MDD. Based on the above findings, we hypothesized that all types of CM are associated with social support.

## Materials and Methods

Data for this study were drawn from the project of the hypothalamic-pituitary-adrenal axis function and magnetic resonance imaging study of trauma-related depression (registration number: ChiCTR1800014591). A detailed description of the design of this project was presented by the previous articles (20, 21).

### Participants

The study enrolled one hundred and sixty-six patients with MDD from the Zhumadian Psychiatric Hospital, Henan, China, from January 2013 to December 2018. Inclusion criteria were as follows: (1) age years ≥ eighteen. (2) diagnosed by two psychiatrists according to the Structured Clinical Interview for the fourth edition of the Diagnostic and Statistical Manual of Mental Disorders (DSM-IV). (3) total score of a 24-item Hamilton rating scale for depression (HAMD-24) > nineteen points. Patients would be excluded if they were satisfied with any of the following criteria: (1) obvious suicide ideation or suicide attempts. (2) current or lifetime severe physical condition. (3) history of any other DSM-IV psychiatric disorders. (4) history of substance dependence or abuse. (5) in the perinatal period.

All participants gave written informed consent. This study was approved by the ethics committee of the Zhumadian Psychiatric Hospital and the ethics committee of the Second Xiangya Hospital of Central South University.

### Measures

#### Childhood Trauma Questionnaire-28 Item Short Form (CTQ-SF)

The CTQ-SF contains 28 self-report items, which are scaled by a five-point Likert ranging from never true (one point) to very often true (five points). Psychiatrists often use this scale to evaluate the five types of CM. Generally, EA refers to the inappropriate emotional response to the child's behavior such as belittling, rejection, and terrorizing (22). PA assesses the violence toward the body of the child by families or caregivers. SA represents sexual contact or forces the child to watch pornography. EN means the deficiency of emotional support (23) and PN is the failure to meet the basic material needs of the child (24). Subjects with CM were defined as ever suffering at least one kind of CM according to the cut-off scores as follows: EA ≥ 13, PA ≥ 10, SA ≥ 8, EN ≥ 15, and PN ≥ 10. The reliability and validity of the Chinese version of CTQ-SF were proved to be good (25).

#### Social Support Rating Scale (SSRS)

The SSRS is a self-report questionnaire in Chinese to assess psychological, objective, and utilization of support. This scale contains ten items and can be divided into three subscales: objective support (three items related to living conditions in the past year, and sources of economical and psychological support in urgent situations), subjective support (four items associated with relationships with colleagues and neighbors, support from family, and numbers of friends who can provide support), and utilization of support (three items in regards to the manner one talks and asks for help when encountering troubles, and the positivity of participation in group activities) (26). The SSRS has good validity and reliability (27, 28).

#### HAMD-24

The HAMD-24 is commonly used by the clinician to rate depressive symptoms. This scale consists of 24 items and the total score range from 0 to 75 points (29). The cut-off scores of HAMD-24 are 8, 20, and 35, which means mild, moderate, and severe depression respectively (30).

#### 14-Item Hamilton Anxiety Rating Scale (HAMA-14)

The HAMA-14 is one of the most widely used rating scales for the severity of anxious symptoms (31). As a clinician-rated scale, it consists of 14 items. Each item is scaled by a five-point Likert ranging from not present (zero point) to severe (four points). This scale has been demonstrated to have good reliability and validity (32).

### Data Analysis

Data analysis was performed on IBM SPSS Statistics 20. Continuous variables with normal distribution were shown as mean ± standard deviation. Those with skewed distribution were shown as median (interquartile range). Pearson and Spearman correlation and partial correlation analysis were performed to assess the relationships between sociodemographic variables, clinical variables, and the SSRS total and subscale scores. Sociodemographic variables related to the SSRS total and subscale scores, respectively, were controlled in Pearson partial correlation analysis. Sociodemographic and clinical variables associated with the SSRS total and subscale scores, respectively, were controlled in Spearman partial correlation analysis. To investigate which associated variables could explain the variation of social support, hierarchical multiple linear regression analysis was adopted. The sociodemographic variables which had statistical significance in correlation analysis were entered in the first step (model 1), and then scores of HAMD-24 or associated types of CM were entered in the second step (model 2). Statistical significance was set as a two-tailed *P* ≤ 0.05.

## Results

### Sociodemographic Information and Clinical Information

As shown in Table 1, patients aged 35 years nearly and the majority of them were married. About ten percent of participants reported a history of smoking and drinking. More than half of patients reported having experienced at least one type of CM. PN and EN were the most prevalent two types of CM.

**Table 1:** Sociodemographic and clinical information (*n* = 166).

**Item**	**Values**
Age (years)	34.91 ± 9.55
Male *N* (%)	73 (44.0)
Married *N* (%)	131 (78.9)
Education (years)	9 (4)
BMI (kg/m^2^)	22.02 ± 3.06
History of smoking *N* (%)	19 (11.4%)
History of drinking *N* (%)	21 (12.7%)
HAMD-24	31.68 ± 7.31
HAMA-14	18.33 ± 6.31
**Reporting CM N (%)**	
Any kind of CM	99 (59.6)
EA	16 (9.6)
PA	15 (9.0)
SA	14 (8.4)
EN	54 (32.5)
PN	83 (50)
**Score of CTQ**	
Total score	40 (18)
EA	7 (3)
PA	5 (2)
SA	5 (0)
EN	12 (8)
PN	9.5 (5)
**Score of SSRS**	
Total score	32.21 ± 7.40
Objective support	7.30 ± 2.47
Subjective support	18.13 ± 5.51
Utilization of support	6.86 ± 1.99

*Data are presented as mean ± standard deviation or median (interquartile range). BMI, Body mass index; HAMD-24, 24-item Hamilton Rating Scale for Depression; HAMA-14, 14-item Hamilton Anxiety Rating Scale; CM, childhood maltreatment; EA, emotional abuse; PA, physical abuse; SA, sexual abuse; EN, emotional neglect; PN, physical neglect; CTQ, Childhood Trauma Questionnaire; SSRS, Social Support Rating Scale*.

### Correlations of Sociodemographic and Clinical Variables With the SSRS Total and Subscale Scores

The results of correlation analyses are shown in Table 2. Age was related to the score of objective support, the score of subjective support, and the SSRS total score (*r* = 0.210, 0.375, and 0.379, respectively). Education and the total score of HAMD-24 were correlated with the score of objective support (*r* = 0.178 and −0.214, respectively). Sex and marriage were associated with the score of subjective support (*r* = 0.163 and −0.217, respectively).

**Table 2:** Correlations of sociodemographic and clinical information with the SSRS total and subscale scores.

**Item**	**SSRS**			
	**Total score**	**Objective support**	**Subjective support**	**Utilization of support**
Age (years)^a^	0.379^**^	0.210^**^	0.375^**^	0.112
Sex^b^	0.110	0.002	0.163^*^	0.004
Marriage^b^	−0.203^**^	−0.089	−0.217^**^	−0.044
Education (years)^b^	−0.071	0.178^*^	−0.135	−0.015
BMI (kg/m^2^)^a^	0.123	0.068	0.114	0.057
History of smoking^b^	−0.003	0.058	−0.030	−0.062
History of drinking^b^	−0.077	0.030	−0.122	0.038
HAMD-24	−0.042^c^	−0.214^c^^,^^**^	−0.041^c^	0.011^a^
HAMA-14	0.004^c^	−0.089^c^	0.054^c^	0.023^a^
EA	−0.231^d^^,^^**^	−0.151^d^	−0.180^d^^,^^^*^^	−0.141^b^
PA	−0.099^d^	0.083^d^	−0.158^d^^,^^^*^^	−0.059^b^
SA	0.072^d^	−0.032^d^	0.069 ^d^	0.083^b^
EN	−0.344^d^^,^^***^	−0.150^d^	−0.327^d^^,^^***^	−0.209^b^^,^^**^
PN	−0.211^d^^,^^**^	−0.120 ^d^	−0.172^d^^,^^^*^^	−0.125^b^

*Bold values indicate statistical significance*;

a *Pearson correlation analysis*;

b *Spearman correlation analysis*;

c *Pearson partial correlation analysis*;

d *Spearman partial correlation analysis*;

* *P_r_ < 0.05*,

** *P_r_ < 0.01*,

*** *P_r_ < 0.001*.

*SSRS, Social Support Rating Scale; BMI, body mass index; HAMD-24, 24-item Hamilton Rating Scale for Depression; HAMA-14, 14-item Hamilton Anxiety Rating Scale; EA, emotional abuse; PA, physical abuse; SA, sexual abuse; EN, emotional neglect; PN, physical neglect*.

Meanwhile, EA, EN, and PN were associated with SSRS total score and the score of subjective support (all *P* < 0.05). Only EN was related to the score of utilization of support (*r* = −0.209, *P* < 0.01). None of the types of CM was correlated with the score of objective support.

### Hierarchical Multiple Linear Regression Analysis of the SSRS Total and Subscale Scores

Table 3 reveals the results of the regression analyses. Age was significant in the final regression models of the SSRS total score, the score of objective support, the score of subjective support (standardized β = 0.282, 0.320, and 0.276, respectively). Education and the total score of HAMD-24 could explain the variation of the score of objective support (standardized β = 0.264 and −0.204, respectively).

**Table 3:** Hierarchical multiple linear regression analysis of the SSRS total and subscale scores.

**Variables**	**Total score**		**Objective support**		**Subjective support**		**Utilization of support**
	**Model 1**	**Model 2**	**Model 1**	**Model 2**	**Model 1**	**Model 2**	**Model**
Age	0.362^***^	0.282^***^	0.296^***^	0.320^***^	0.348^***^	0.276^***^	
Marriage	−0.058	−0.065			−0.047	−0.058	
Education			0.280^***^	0.264^**^			
Sex					0.089	0.083	
HAMD-24				−0.204^**^			
EA		−0.089				−0.030	
EN		−0.357^***^				−0.339^***^	−0.205^**^
PN		0.081				0.137	
PA						−0.117	
Total R^2^	0.147^***^	0.267^***^	0.115^***^	0.156^***^	0.151^***^	0.255^***^	0.042^**^

*Data are presented as standardized β; Bold values indicate statistical significance*;

** *P < 0.01*,

*** *P < 0.001*.

*SSRS, Social Support Rating Scale; EA, emotional abuse; EN, emotional neglect; PN, physical neglect; PA, physical abuse*.

EN had statistical significance in the final regression models of SSRS total score, the score of subjective support, and the score of utilization of support (standardized β = −0.357, −0.339, and −0.205, respectively; R^2^ = 0.267, 0.255, and 0.042, respectively).

## Discussion

To the best knowledge, the present study is the first one to investigate the associations of different types of CM with social support in Chinese patients with MDD. We found that in Chinese patients with MDD: (1) EN and PN were the most prevalent two types of CM. (2) in regression models, only EN was associated with social support. Our findings highlight the significance of EN in impairing social support in Chinese patients with MDD.

### High Prevalence of CM in Patients With MDD

In the present study, the prevalence of CM is lower than that in a German MDD sample (74.7%) (33), while higher than that in another Chinese MDD sample (48.8%) (34). Meanwhile, in this Chinese study, they reported higher prevalence of PN, EN, and EA in the MDD group among types of CM (34), which is consistent with us. It indicates the three types of CM possibly have close relationships with MDD, different from foreign studies holding that abuse is more related to depression (23).

### EN Is Associated With Low Social Support in Patients With MDD

In China, about twenty-six percent of children ever experience childhood neglect, which is higher compared with other countries (35) and leads to large economic losses (35). However, little do we know about the impact of childhood neglect on the mental health of the Chinese (36). In contrast to our hypothesis, we reported only a negative association of EN with social support, which is consistent with the existing literature. A study examining the influence of CM on the adaption of soldiers to army life found that EN has a special negative impact on the access to social support from units (37). Harmer et al. (16) investigated 46 adults recovering from addiction and reported that severer neglect is associated with lower social support from the family in Australia.

The exact mechanisms under the association of EN with social support were unknown. We supposed the probable reasons are as follows. Firstly, EN was one of the most prevalent types of CM in this study, which makes it easier to have a relationship with social support. In addition, the correlated coefficients of EN with social support were the highest among the five types of CM, making the relationships between other types of CM and social support are covered by EN. Moreover, the experience with EN makes one person is not good at expressing emotion (38) and feel not being loved, valued, and cared for, which lets him hold that other people will refuse him and tend to less utilize social support. Meanwhile, survivors of EN have deficits in the development of intimate relationships (39), resulting in a weak supportive network insufficient for them to use. Finally, there may exist a mediator between EN and social support, such as attachment (40). According to Kapeleris and Paivio (38), EN is correlated with emotional competence such as lack of sense of contentment, worth, and importance, which contributes to insecure attachment styles leading to low trust in others and poor utilization of social support. The reason why EN was not associated with objective support was unclear. However, it indicates that the correlations of age, education, and severity of depression with objective support were more significant than EN in the sample of the present study.

Social support is associated with the elevated risk (2), severity (41–43), course (44), and outcomes (45) of depression, and even mediates the correlation of depression with suicidal ideation (46). The higher proportion of EN (47, 48) and its significant association with social support indicate that it should be taken into consideration in studies involved with social support in patients with MDD. Psychotherapy mitigating the potentially detrimental effects of EN on social support may be effective in the prevention and recovery of depression.

### Limitations

Despite the advantages of this study, several limitations should be considered when interpreting these findings. Firstly, the data of CM was collected retrospectively, which could induce recall bias in the assessment. Prospective research should be considered in the future. Secondly, CTQ-SF and SSRS are self-report scales vulnerable to subjective effects, especially in patients with MDD. Depressed patients tend to exaggerate their adverse experiences in childhood and misjudge social support in the present life. Thirdly, variables such as family structure, economic status, and individual occupations correlated with social support were failed to be enrolled and controlled in the current study, which should be considered in the future. Finally, the explanatory effects of the regression models on social support are relatively unsatisfactory. Future studies should include more related variables to build models with better effects.

### Conclusions

PN and EN are the most prevalent types of CM in Chinese patients with MDD. EN is the only type of CM related to social support in regression analyses. Studies associated with the social support system in Chinese patients with MDD in the future should take the assessment of EN into account. Moreover, developing special interventions to EN may help to improve social support and contribute to the mental health of Chinese patients with MDD.

## Data Availability Statement

The raw data supporting the conclusions of this article will be made available by the authors, without undue reservation.

## Ethics Statement

The studies involving human participants were reviewed and approved by the Ethics Committee of the Zhumadian Psychiatric Hospital and the Second Xiangya Hospital of Central South University. The patients/participants provided their written informed consent to participate in this study.

## Author Contributions

LL and BL co-designed the topic. MW, XL, JS, QD, LZ, JL, YJ, PW, HG, FZ, YZ, and BL are responsible for participant recruitment and data collection. XQ undertook the statistical analyses and wrote the initial draft of the manuscript. BL contributed substantial revisions to the manuscript. All authors contributed to the article and approved the submitted version.

## Funding

This study was supported by the National Science and Technologic Program of China (2015BAI13B02), the Defense Innovative Special Region Program (17-163-17-XZ-004-005-01), the National Natural Science Foundation of China (81171286, 91232714, and 81601180). The funding sources had no role in the study design, data collection and analysis, interpretation of the data, preparation and approval of the manuscript, and decision to submit the manuscript for publication.

## Conflict of Interest

The authors declare that the research was conducted in the absence of any commercial or financial relationships that could be construed as a potential conflict of interest. The reviewer LN declared a shared affiliation with several of the authors XQ, MW, XL, JS, QD, LZ, JL, YJ, BL, YZ and LL to the handling editor at the time of the review.

## Publisher's Note

All claims expressed in this article are solely those of the authors and do not necessarily represent those of their affiliated organizations, or those of the publisher, the editors and the reviewers. Any product that may be evaluated in this article, or claim that may be made by its manufacturer, is not guaranteed or endorsed by the publisher.
